# [Corrigendum] Long non‑coding RNA H19 regulates LASP1 expression in osteosarcoma by competitively binding to miR‑29a‑3p

**DOI:** 10.3892/or.2024.8737

**Published:** 2024-04-17

**Authors:** Hao Jin, Huan Wang, Xin Jin, Wenbo Wang

Oncol Rep 46: 207, 2021; DOI: 10.3892/or.2021.8158

Following the publication of the above article, an interested reader drew to the authors' attention that, for the cell invasion assay experiments shown in Fig. 2D on p. 5, there appeared to be an overlapping section of data comparing between the Sao-2/Control and MG-63/siH19 panels, such that these data had been derived from the same original source where the panels were intended to portray the results from differently performed epxeriments. Upon examining their original data, the authors have realized that, in Fig. 2D, an inadvertent error was made in the copying and pasting of the two groups of pictures, resulting in the image belonging to the Saos-2 cell experiment being mistakenly pasted as the image for the MG-63 cell experiment. The authors carefully checked the original pictures and the experimental record, and found that the two groups of cells were close to the same morphology.

The corrected version of Fig. 2, containing data from an alternatively performed experiment for Fig. 2D, is shown on the next page. Note that the error did not affect the overall conclusions reported in the paper. All the authors agree with the publication of this corrigendum, and are grateful to the Editor of *Oncology Reports* for allowing them the opportunity to publish this. They also apologize to the readership for any inconvenience caused.

## Figures and Tables

**Figure 2.**
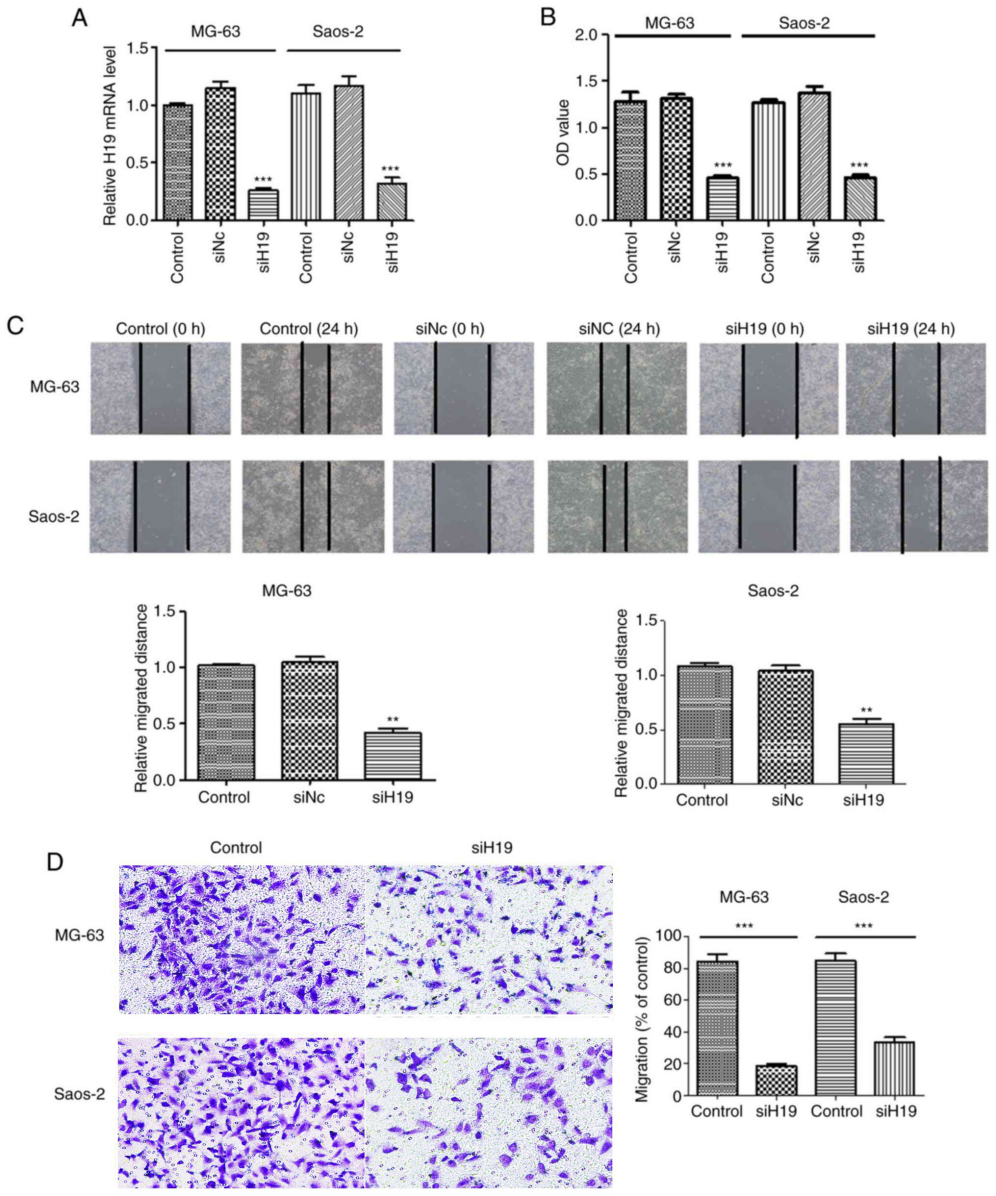
H19 siRNA inhibits proliferation, migration and invasion of osteosarcoma cells. (A) H19 siRNA was successfully transfected into MG-63 and Saos-2 cells. (B) H19 siRNA decreased the proliferative capacity of MG-63 and Saos-2 cells, as shown by Cell Counting Kit-8 assay. OD values (450 nm) were quantitated. H19 siRNA decreased (C) migration and (D) invasion of MG-63 and Saos-2 cells vs. control and siNC groups. Magnification, ×100. Scale bar, 50 µm. Data are expressed as the mean ± SEM (n=3). **P<0.01 and ***P<0.001 vs. control. si, small interfering; OD, optical density; NC, negative control.

